# Resveratrol Induces Vascular Smooth Muscle Cell Differentiation through Stimulation of SirT1 and AMPK

**DOI:** 10.1371/journal.pone.0085495

**Published:** 2014-01-08

**Authors:** Anne Marie Thompson, Kathleen A. Martin, Eva M. Rzucidlo

**Affiliations:** 1 Section of Vascular Surgery, Dartmouth Medical School, Lebanon, New Hampshire, United States of America; 2 Section of Cardiovascular Medicine, Yale School of Medicine, New Haven, Connecticut, United States of America; University of Maryland, United States of America

## Abstract

Phenotypic plasticity in vascular smooth muscle cells (VSMC) is necessary for vessel maintenance, repair and adaptation to vascular changes associated with aging. De-differentiated VSMC contribute to pathologies including atherosclerosis and intimal hyperplasia. As resveratrol has been reported to have cardio- protective effects, we investigated its role in VSMC phenotypic modulation. We demonstrated the novel finding that resveratrol promoted VSMC differentiation as measured by contractile protein expression, contractile morphology and contraction in collagen gels. Resveratrol induced VSMC differentiation through stimulation of SirT1 and AMPK. We made the novel finding that low or high dose resveratrol had an initially different mechanism on induction of differentiation. We found that low dose resveratrol stimulated differentiation through SirT1-mediated activation of AKT, whereas high dose resveratrol stimulated differentiation through AMPK-mediated inhibition of the mTORC1 pathway, allowing activation of AKT. The health effects of resveratrol in cardiovascular diseases, cancer and longevity are an area of active research. We have demonstrated a supplemental avenue where-by resveratrol may promote health by maintaining and enhancing plasticity of the vasculature.

## Introduction

Vascular smooth muscle cells (VSMCs) are not terminally differentiated; they are capable of responding to both intrinsic and extrinsic vessel signaling and can modify their phenotype in physiological and pathophysiological settings. This capacity for reversible phenotypic switching is a property of a mature VSMC, where the primary function is to regulate blood vessel diameter by contracting or relaxing. Differentiated VSMCs proliferate slowly, have low synthetic activity and express a specific set of contractile proteins [1], [2]. Growth factor stimulation in vitro, or in response to injury *in vivo*, elicits a phenotype change; VSMC de-differentiate, express reduced levels of contractile proteins, proliferate and can migrate to the site of injury where they participate in vascular repair and remodeling. The plasticity of VSMC creates an adaptive, multifunctional smooth muscle layer within the artery. The capacity for phenotypic modulation is a necessity for maintenance and repair of the vessel in response to environmental insults.

Several signaling pathways control phenotypic modulation. These pathways are connected by reciprocal activation or inhibition. Several key signaling molecules have been implicated in differentiation, including mTORC1, AKT and AMPK [3], [4]. The mTORC1 pathway controls cell size, primarily by altering protein synthesis. Growth factor activation of this pathway promotes VSMC de-differentiation and proliferation, while mTORC1 inhibition promotes differentiation, by relieving the classical S6K1-mediated negative feedback regulation of insulin receptor substrate-1 (IRS-1) signaling to PI3K/AKT [3]. The AKT pathway controls cellular metabolism, growth and apoptosis [5]. We have previously demonstrated that AKT2 activation was essential for VSMC differentiation [3]. Furthermore, we have demonstrated that the AMPK signaling pathway, which controls cellular energy homeostasis, inhibits mTORC1 and thereby promotes differentiation through activation of AKT [4]. These differentiation and de-differentiation promoting molecules are intimately intertwined and their activation or inhibition is dynamically influenced by vessel homeostasis.

Resveratrol is a polyphenol found in the skin of grapes, berries and peanuts that can activate AMPK and sirtuins [6], [7]. Resveratrol possess antioxidant properties [6], increases nitric oxide synthase production [8] and improves mitochondrial function [6]–[9] by activating AMPK and sirtuins.

Sirtuins are a family of proteins that function as NAD^+^ dependent deacetylases and as such are tied to the metabolic state of the cell. There are seven mammalian sirtuins (SirT1-7), each functioning in a distinct capacity, but ultimately tied to cellular metabolism. SirT1 is the mammalian homologue of Sir2, which was first identified in *Saccharomyces cerevisiae* [10], and is recognized for its association with longevity [11]–[13].

In the current study, we investigated the effect of resveratrol on promotion of VSMC differentiation in human VSMC (HuVSMC). We tested the hypothesis that resveratrol treatment increases levels of SirT1, which promotes VSMC differentiation. We made the novel finding that low or high dose resveratrol had a differential mechanism on induction of differentiation. We found that low dose resveratrol stimulated differentiation through SirT1-mediated activation of AKT, whereas high dose resveratrol stimulated differentiation through AMPK-mediated inhibition of the mTORC1 pathway, allowing activation of AKT.

## Materials and Methods

### Materials

Antibodies against SirT1, phosphorylated ribosomal protein-S6 (Ser240/244), AMPK-α, phosphorylated AMPK-α (Thr172) and phosphorylated AKT (Ser473) were purchased from Cell Signaling (Beverly, MA). Antibodies against β-tubulin and phosphorylated p70S6K (Thr421/Ser424) were purchased from Santa Cruz Biotechnology (Santa Cruz, CA). GAPDH antibody was purchased from Abcam (Cambridge, MA). Antibodies against smooth muscle myosin heavy chain (SM2-MHC) and calponin along with resveratrol and carbachol were purchased from Sigma-Aldrich (St. Louis, MO). Pierce BCA protein assay kit was purchased from Thermo Scientific (Rockford, IL). API-2 was purchased from EMB Millipore (Darmstadt, Germany).

### Cell Culture

Human vascular smooth muscle cells (HuVSMC) were purchased from Lifeline Cell Technology (Frederick, MD; FC-0031) or isolated from vascular surgery patients or organ donor tissue. The Dartmouth College Committee for the Protection of Human Subjects reviewed the study project in detail. As the specimens were to be destroyed and the cells are not immortalized (destroyed after 10 passages), neither patient consent nor Institutional Review Board approval was required. Tissues obtained from patients or donors included renal artery, tibial artery and femoral artery. Tissue was received in sterile saline. Outer adventitial layer was dissected from medial layer and cells were prepared from the medial layer using the explant method in M199 media with 10% fetal bovine serum, penicillin, streptomycin, and L-glutamine. The cells were verified as smooth muscle cells through staining of smooth muscle specific markers and the lack of staining for FSP1, a fibroblast specific marker. VSMCs at passages 2–7 were used for experiments. Data in each experiment have been confirmed in VSMCs from at least two different preparations. Cells were grown to 70–80% confluence and 24 hours prior to resveratrol or control treatment, cells were transferred to media containing 2.5% fetal bovine serum for the duration of the experiment. Control treatment was the vehicle utilized in the formulation of the drug treatment.

### Morphology

HuVSMC were plated on coverslips. The same numbers of cells were added to each coverslip. Cells were treated with 0–100 µM resveratrol, in duplicate, for 24 hours. Cells were washed with PBS, fixed with acetone/methanol (1∶1) for 10 minutes and then washed with PBS. Next, cells were stained with coomassie blue for 10 minutes and washed with PBS. Cover slips were allowed to air dry and then were mounted on slides. Images of the cells were captured using SPOT™ 5.0 software. Quantification of the total cell area (µm^2^) at 40X magnification was obtained by measureing three areas per duplicate treatment and then averaging the areas.

### Immuno-Flourescence

HuVSMC were plated on coverslips. The same numbers of cells were added to each coverslip. Cells were treated with 3 µM resveratrol or control, in triplicate, for 24 hours. Coverslips were washed with PBS and fixed acetone/methanol (1∶1) for 10 minutes. Next, coverslips were washed twice with TBS/0.1%Tween 20. Coverslips were blocked with 3% BSA in TBS/0.1%Tween 20 for 1 hour. Primary antibody (SM2-MHC or SM-Alpha Actin, 1∶100) were added to coverslips and incubated overnight at 4°C. Primary antibodies were removed from coverslip and washed three times in TBS/0.1%Tween 20. Secondary antibodies (Alexa Fluor 568 Anti-Rabbit or Alexa Fluor 488 Anti-Mouse) were added to the coverslips for 1 hour. The secondary antibodies were removed and the coverslips were washed three times in TBS/0.1%Tween 20. DAPI stain was added for 5 minutes and the coverslips were washed with TBS/0.1%Tween 20. Coverslips were mounted on slides and analyzed on Zeiss LSM 510 Meta confocal microscope.

### *In Vitro* Contraction Assay

HuVSMC were pre-treated with 3–5 µM resveratrol or control for 24 hours prior to being trypsinized for experimentation. Twenty-four well plates were coated with 3% BSA for 1 hour. A suspension of Type I collagen (1 ng/mL), M199 media with 2.5% fetal bovine serum and pre-treated VSMCs was prepared. 400 mL of cell/collagen suspension was pipetted into the BSA-coated plates and incubated at 37°C for 1 hour. 1×10^5^ VSMCs were plated per collagen disc. Collagen discs were gently detached from sides of well. Detached collagen discs were re-treated with the same treatment that was administered prior to being suspended in collagen and photographed over a time course. Area of collagen discs was assessed using ImageJ.

### Organ Culture Model

Human tibial arteries were received in sterile saline, placed in sterile cell culture dishes and cross-sectioned into rings 2 mm in thickness. Tissue was maintained in M199 media with 10% fetal bovine serum with or without resveratrol treatment. Media was changed every other day until collected for western blot analysis.

### Western Blot Analysis

Cells were washed with Hanks buffer and scraped in 50–100 µL of lysis buffer (with protease inhibitors), centrifuged, and the supernatant was collected. Protein content was determined by BCA protein assay. Total cell extracts containing 16–20 µg of protein were prepared in SDS sample buffer and subjected to SDS-PAGE and western blot analysis. Proteins were transferred to nitrocellulose membranes prior to immunodetection.

### Transient Transfection of siRNA and Plasmid

Transient transfection of small interfering RNA (siRNA) was performed via Nucleofector (Lonza Walkersville Inc., MD) as previously described [3]. For gene knockdown, 500,000–1,000,000 cells were transfected with 8 µg siRNA and cultured in 2.5% fetal bovine serum for 24–48 hours. Cells were then treated with resveratrol for 24 hours and harvested for western blot. SirT1 siRNA (sc-40986) was purchased from Santa Cruz Biotechnology (Santa Cruz, CA). Control siGENOME non-targeting siRNA (D-001206-13-05) was purchased from Thermoscientific (Pittsburg, PA) for use with the SirT1 siRNA experiments. AMPKα1 siRNA (SI02622235) and a matching nonsilencing control siRNA were purchased from Qiagen (Germantown, MD).

Transfection of SirT1 plasmid (#13812, Addgene, Cambridge MA) was performed via Nucleofector as previously described [3]. For gene overexpression, 500,000–1,000,000 cells were transfected with 8 µg SirT1 plasmid and cultured in 2.5% fetal bovine serum for 24–48 hours. Cells were treated with resveratrol for 24 hours and harvested for western blot analysis.

### Statistical Analysis

Results are expressed as mean±SEM. Statistical differences between groups were determined with a one-way analysis of variance (ANOVA) with Newman-Keuls post-hoc test using GraphPad Prism software. A p value less than 0.05 was considered significant.

## Results

### Resveratrol Induces Differentiation in Human Vascular Smooth Muscle Cells

To determine the effect of resveratrol on differentiation, we used HuVSMC isolated from multiple donors and various vascular beds, which were maintained in culture conditions. HuVSMC cultured in serum are de-differentiated; they express low levels of contractile proteins, they produce increased extracellular matrix, they have a fibroblast-like morphology and they are proliferative. Measurement of contractile protein expression, evaluation of morphology and the capacity to contract are standard methods that have been used to characterize vascular smooth muscle cell phenotype [3], [4].

**Figure 1:**
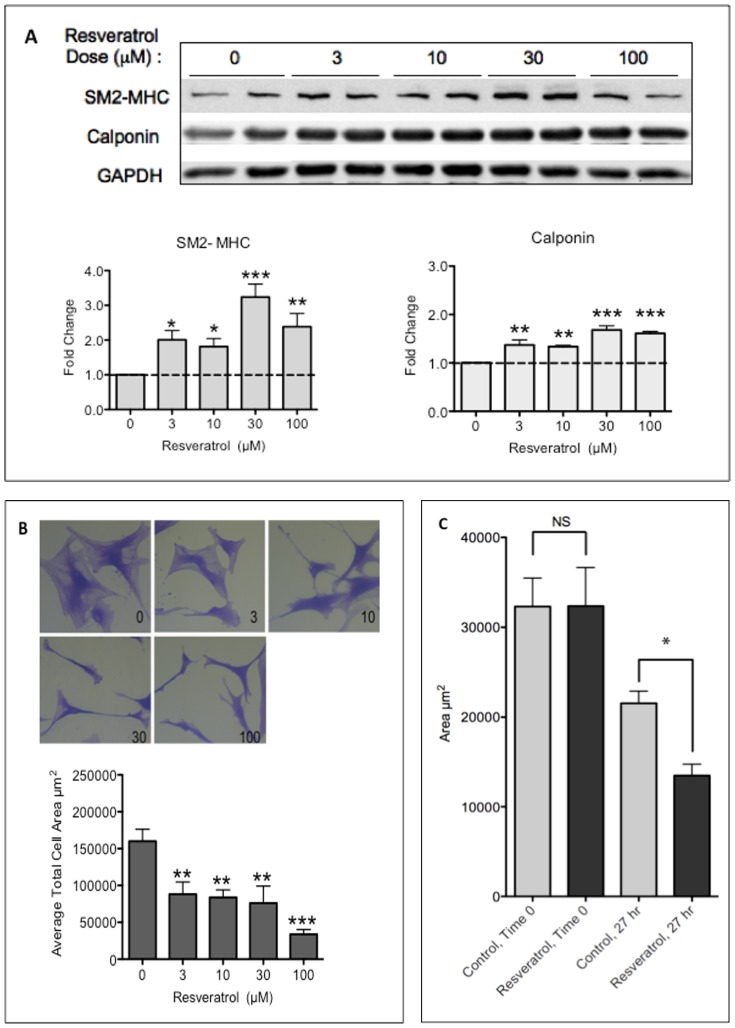
Resveratrol Induces Differentiation in HuVSMC. A, HuVSMC treated with increasing doses of resveratrol for 24 hours prior to western blot assessment with primary antibodies as indicated. Bar graphs represent densitometric quantification. N = 5 experiments. Probability values are indicated above bars: * p<0.05, **p<0.01, ***p<0.001 versus control. B, Cell morphology after resveratrol treatment. HuVSMC treated with 0, 3, 10, 30 and 100 µM resveratrol. 40X magnification. Bar graphs represent mean area change plus standard error of mean. N = 6, Probability values are indicated above bars: **p<0.01, ***p<0.001 versus control. C, HuVSMC pretreated with 3 µM resveratrol for 24 hours contracted the collagen disc, compared to control. Bar graphs represent mean area change plus standard error of mean. N = 2 experiments, in triplicate. Probability values are indicated above bars: * p<0.05 versus control.

We first performed a dose escalation experiment and monitored markers of differentiation. HuVSMC were treated with 3–100 µM resveratrol or control for 24 hours. Resveratrol treatment, at all doses, induced expression of protein markers of differentiation, including smooth muscle myosin heavy chain (SM2-MHC), the most stringent marker of VSMC differentiation [1], and calponin (Figure 1A).

Differentiated HuVSMC can also be qualified by using characteristic changes in morphology. Differentiated HuVSMC are spindle shaped with a reduced cellular area, when compared to the large, fibroblast-like morphology of de-differentiated VSMC. Resveratrol treatment (3–100 µM), promoted a change in cellular morphology to the contractile phenotype with a statistically significant decrease in two-dimensional cellular area (Figure 1B). We further verified the results of the two-dimension morphology analysis using immuno-fluorescence staining for VSMC markers SM-Alpha Actin and SM2-MHC, which demonstrate diffuse staining of contractile proteins with control treatment and organized staining of contractile proteins with resveratrol treatment (Figure S1).

Contractile capacity is a powerful functional measure of VSMC phenotype. Differentiated VSMC demonstrate an increased capacity for *in vitro* contraction [14]. Resveratrol, at the minimum effective dose of 3–5 µM, significantly increased HuVSMC contraction in collagen discs, compared to control (Figure 1C). We confirmed that resveratrol-induced contraction took place in a time frame suggestive of protein up regulation and that it was as effective as carbachol, a positive control for VSMC contraction [15] (Figure S2). These combined data demonstrate that resveratrol promoted a differentiated contractile phenotype in cultured HuVSMC.

### SirT1 Induces Changes in Contractile Proteins Indicative of Differentiation

**Figure 2:**
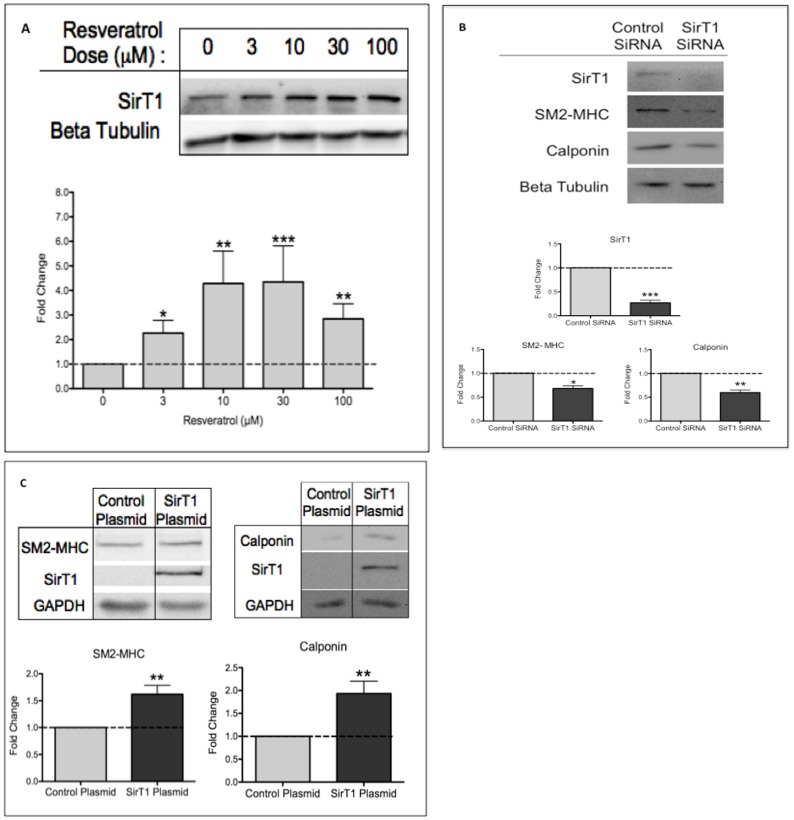
SirT1 Induces Changes in Contractile Proteins Indicative of Differentiation. A, HuVSMC treated with increasing concentrations of resveratrol for 24 hours before western blot assessment with primary antibodies as indicated. Bar graphs represent densitometric quantification. N = 9 experiments. Probability values are indicated above bars: * p<0.05, **p<0.01, ***p<0.001 versus control. B, HuVSMC were transfected with control small interfering (si) RNA or SirT1 siRNA for 48 hours and then assessed by western blot with primary antibodies as indicated. Bar graphs represent densitometric quantification. N = 2 experiments, in duplicate. Probability values are indicated above bars: * p<0.05, **p<0.01 versus control. C, HuVSMC were transfected with control or SirT1 plasmid for 48 hours and then assessed by western blot with primary antibodies as indicated. At higher exposures, endogenous SirT1 is present in this experiment (data not shown). Bar graphs represent densitometric quantification. N = 3 experiments. Probability values are indicated above bars: **p<0.01 versus control.

As resveratrol induces a wide range of effects in endothelial, epithelial and fibroblastic cells [6], we determined whether resveratrol altered SirT1 protein expression in HuVSMC. We found that resveratrol (3–100 µM) significantly increased SirT1 protein expression in HuVSMC (Figure 2A). To determine if SirT1 was necessary and sufficient for VSMC differentiation, we performed knockdown and overexpression experiments. We demonstrated that a 70% knockdown of endogenous SirT1 using siRNA significantly reduced basal levels of SM2-MHC and calponin contractile protein expression (Figure 2B). Furthermore, we demonstrated that upon SirT1 overexpression, SM2-MHC and calponin were significantly increased from the de-differentiated baseline state (Figure 2C). As a note, endogenous SirT1 is visualized at higher exposures. Together, these data indicate that SirT1 expression is necessary and sufficient for contractile protein expression in cultured HuVSMC.

### Mechanism of Low Dose Resveratrol Induced Contractile Protein Expression

**Figure 3:**
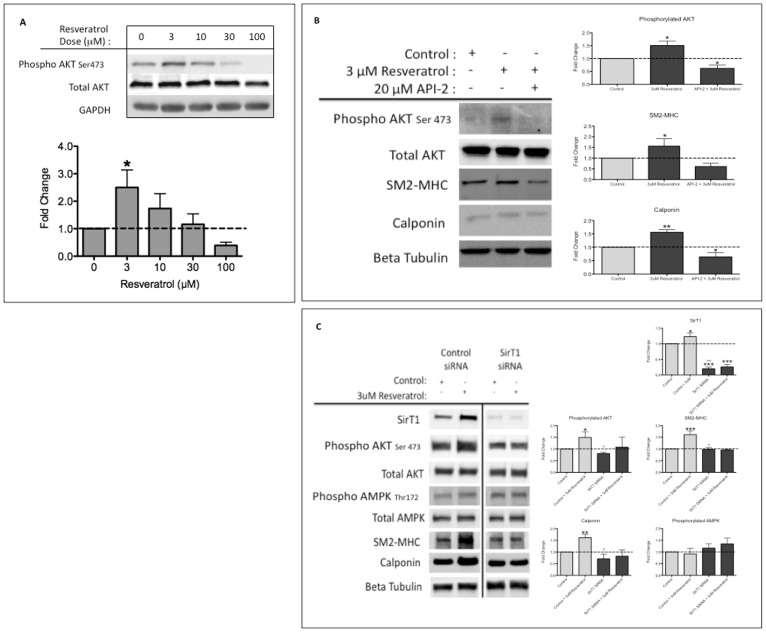
Resveratrol Induced Differentiation in VSMC via SirT1-AKT Signaling. A, HuVSMC treated with increasing concentrations of resveratrol for 24 hours before western blot assessment with primary antibodies as indicated. Bar graphs represent densitometric quantification. N = 9 experiments. Probability values are indicated above bars: * p<0.05 versus control. B, HuVSMC were treated with 20 µM API-2 one hour prior to resveratrol or control treatment. Twenty-four hours following resveratrol treatment, cells were collected and assessed by western blot with primary antibodies as indicated. Bar graphs represent densitometric quantification. N = 4 experiments. Probability values are indicated above bars: * p<0.05, **p<0.01 versus control. C, HuVSMC were transfected with control siRNA or SirT1 siRNA for 24 hours, prior to treatment with 3 µM resveratrol or control. Twenty-four hours after treatment, cells were collected and assessed by western blot with primary antibodies as indicated. Bar graphs represent densitometric quantification. N = 3 experiments, in duplicate. Probability values are indicated above bars: * p<0.05, **p<0.01, *** p<0.001 versus control or <p<0.01, <<p<0.001 versus control+resveratrol.

In an effort to determine how SirT1 participates in resveratrol- induced contractile protein expression, we first examined whether resveratrol activated the AKT pathway as we have previously demonstrated that AKT2 activation was essential for VSMC differentiation [3]. We found, with low dose resveratrol treatment (3 µM), the AKT pathway was activated as demonstrated by an increase in AKT phosphorylated at Ser473 (Figure 3A). Of note, at the 24-hour time point, we did not observe significant AKT activation with high dose resveratrol. Additionally, we verified, using an isoform specific immunoprecipitation, that AKT2 was the isoform activated following resveratrol treatment (Figure S3). Next, in an effort to confirm that AKT activation was necessary for low dose resveratrol-induced differentiation, we utilized a specific inhibitor of AKT activation, API-2 [16]. We treated HuVSMC with and without low dose resveratrol in the presence or absence of API-2. We found that in the absence of AKT activation, 3 µM resveratrol treatment was unable to induce differentiation, as demonstrated by a lack of change in contractile protein expression (Figure 3B). In addition, we verified that inhibition of AKT activation prevented differentiation with low or high dose resveratrol, indicating that AKT activation is necessary for induction of differentiation with low or high dose resveratrol (Figure S4).

Finally, to further verify the mechanism by which low dose resveratrol induces differentiation, we conducted a SirT1 knockdown experiment in HuVSMC. Having demonstrated that that low dose resveratrol, at 24 hours post treatment, activates AKT and that high dose resveratrol, at 24 hours post treatment, did not significantly induce AKT activation; we treated HuVSMC with low dose resveratrol (3 µM) for 24 hours, after SirT1 knockdown. We found that resveratrol treatment, in the control siRNA group induced differentiation through stimulation of SirT1 and activation of AKT, as measured by an increase in contractile protein, SirT1 expression and AKT activation. However, with an 80% knockdown of SirT1, low dose resveratrol treatment did not induce differentiation, as measured by lack of change in contractile protein expression and absence of AKT activation. (Figure 3C). Together, these data demonstrate that low dose resveratrol stimulates SirT1, which is required for activation of AKT and the induction of differentiation. Intriguingly, resveratrol treatment above 3 µM did not significantly activate AKT, suggesting that resveratrol treatment above 3 µM may have additional activity that is independent of SirT1 activation of AKT.

### Mechanism of High Dose Resveratrol Induced Contractile Protein Expression

We have demonstrated VSMC differentiation at higher dose resveratrol treatment that is independent of SirT1 activation of AKT. Provided that resveratrol is known to induce AMPK activation [9], [17], we hypothesized that high dose resveratrol induces differentiation through AMPK activation.

**Figure 4:**
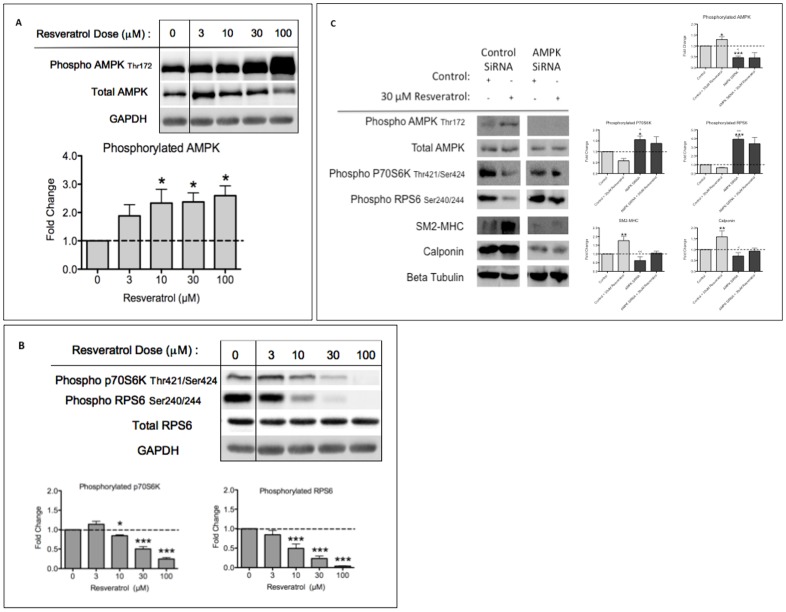
AMPK Inhibits the mTORC1 Pathway to Induce Differentiation. A, HuVSMC treated with increasing concentrations of resveratrol for 24 hours prior to western blot assessment with primary antibodies as indicated. Bar graphs represent densitometric quantification. Samples have been normalized to GAPDH and then fold change from normalized values have been graphed. N = 5 experiments. Probability values are indicated above bars: * p<0.05 versus control. B, HuVSMC treated with increasing concentrations of resveratrol for 24 hours prior to western blot assessment of primary antibodies as indicated. Bar graphs represent densitometric quantification. Samples have been normalized to GAPDH and then fold change from normalized values have been graphed. N = 3 experiments. Probability values are indicated above bars: * p<0.05, *** p<0.001 versus control. C, HuVSMC were transfected with control siRNA or AMPK α1 siRNA for 24 hours, prior to treatment with 30 µM resveratrol or control. Twenty-four hours after treatment, cells were collected and assessed by western blot with primary antibodies as indicated. Bar graphs represent densitometric quantification. N = 4 experiments. Probability values are indicated above bars: * p<0.05, **p<0.01, *** p<0.001 versus control or <p<0.01, <<p<0.001 versus control+resveratrol.

We treated HuVSMC with resveratrol (3–100 µM) for 24 hours and demonstrated that resveratrol treatment induced AMPK activity as measured by AMPK phosphorylated at Thr172 (Figure 4A). We have previously shown that AMPK activation induces HuVSMC differentiation via inhibition of the mTORC1/S6K1 pathway, which allows activation of AKT [4]. We found that resveratrol induced AMPK activity correlated with an inhibition of the mTORC1 pathway as measured by phosphorylation of the mTORC1 substrate p70S6K and its substrate RPS6 (Figure 4B). Consistent with our hypothesis, resveratrol treatment at 3 µM did not significantly activate AMPK or inhibit mTORC1, suggesting that resveratrol treatment at this low dose is acting independent of AMPK.

Finally, to further verify the mechanism by which high dose resveratrol induces differentiation, we conducted an AMPK α1 knockdown experiment in HuVSMC.

Having demonstrated that low dose resveratrol does not significantly activate AMPK or inhibit the mTORC1 pathway (Figure 4A,B); we treated HuVSMC with high dose resveratrol (30 µM) for 24 hours, after AMPK α1 knockdown. We found that resveratrol treatment, in the control siRNA group induced differentiation through activation of AMPK and subsequent inhibition of mTORC1, as measured by an increase in contractile protein expression, AMPK activation and a decrease in phosphorylation of the mTORC1 substrate p70S6K and its substrate RPS6. However, with a 50% knock down of AMPK α1, high dose resveratrol treatment did not induce differentiation, as measured by a lack of change in contractile protein expression and absence of mTORC1 pathway inhibition (Figure 4C). Additionally, we verified, using AMPK α1 siRNA with low and high dose resveratrol that in the absence of AMPK α1, low dose resveratrol does induce a marker of differentiation, whereas high dose does not; verifying that high dose resveratrol works through AMPK (Figure S5).

Together, our data demonstrates the initial mechanism by which resveratrol induces differentiation in HuVSMC is dependent on the dose of resveratrol that is administered. Low dose resveratrol (3 µM) induces differentiation through stimulation of SirT1 and activation of AKT, whereas high dose resveratrol (30 µM) induces differentiation through stimulation of AMPK and inhibition of the mTORC1 pathway, allowing activation of AKT.

### Resveratrol Induced Differentiation in Intact Tissue

**Figure 5:**
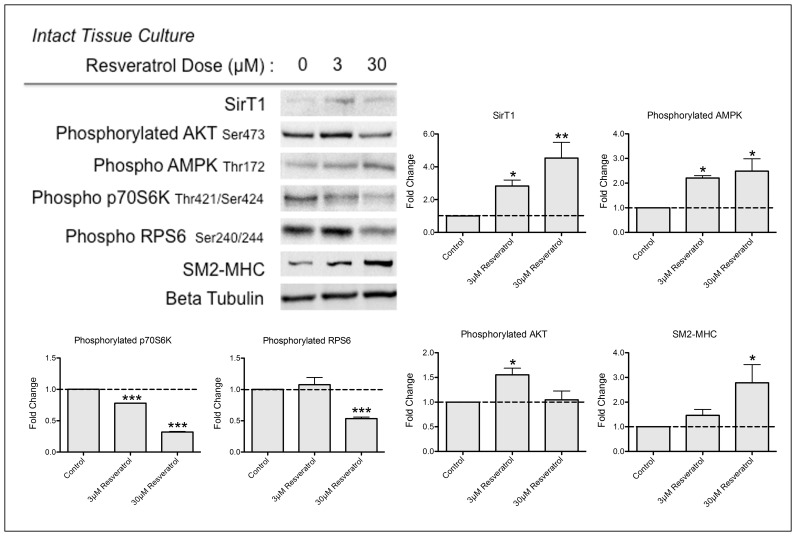
Resveratrol-Induced Differentiation in Whole Tissue. Tibial artery rings cultured for 10 = 3 experiments. Probability values are indicated above bars: *p<0.05, *** p<0.001, versus control.

We have demonstrated that both SirT1 and AMPK are stimulated by resveratrol and participate in the induction of differentiation in cultured HuVSMC. To verify that these signaling proteins play a role in phenotypic modulation in intact vascular tissue, we utilized an organ culture model. In this model, smooth muscle cells in the medial layer de-differentiate when the vessel is cultured in media with serum growth factors. Intact rings of human artery were cultured in media with 10% fetal bovine serum with or without resveratrol for 10 days. Resveratrol treatment elicited changes in contractile and signaling proteins consistent with the results from our *in vitro* cell culture (Figure 5). Low dose resveratrol treatment (3 µM) significantly increased SirT1 and activated AKT. High dose resveratrol treatment (30 µM) significantly increased SirT1, activated AMPK, inhibited the mTORC1 pathway as measured by phosphorylation of the mTORC1 substrate p70S6K and its substrate RPS6 and increased SM2-MHC. The result of this intact vessel model confirms our findings from isolated smooth muscle cells; resveratrol stimulates SirT1 and AMPK to induce differentiation.

## Discussion and Conclusions

Vascular smooth muscle cell differentiation is a vital process in the maintenance and repair of the vasculature. Loss of vascular smooth muscle cell plasticity is one component in the progression of age-related vasculature deterioration. We have made the novel finding that resveratrol induced differentiation through a dose dependent mechanism. Low dose resveratrol induced differentiation through stimulation of SirT1, which activated AKT to induce differentiation. High dose resveratrol induced differentiation through activation of AMPK, which inhibited the mTORC1 pathway and relieved AKT inhibition, therefore-promoting differentiation. Despite the dose of resveratrol utilized, the final downstream effector for resveratrol-induced differentiation is AKT.

Sirtuins are a family of enzymes that improve metabolic function, which is intimately tied to protection against age-related deterioration. Sirtuins control transcription factors associated with decreasing inflammation, increasing fatty acid oxidation, increasing insulin sensitivity, mediating DNA repair and altering mitochondria during energy adaptation [8], [11], [13], [18].

AMPK is the master regulator of energy metabolism; it matches energy use to energy demand by altering glycogen utilization, carbohydrate and lipid metabolism, fatty acid uptake and oxidation, and protein synthesis [19].

**Figure 6:**
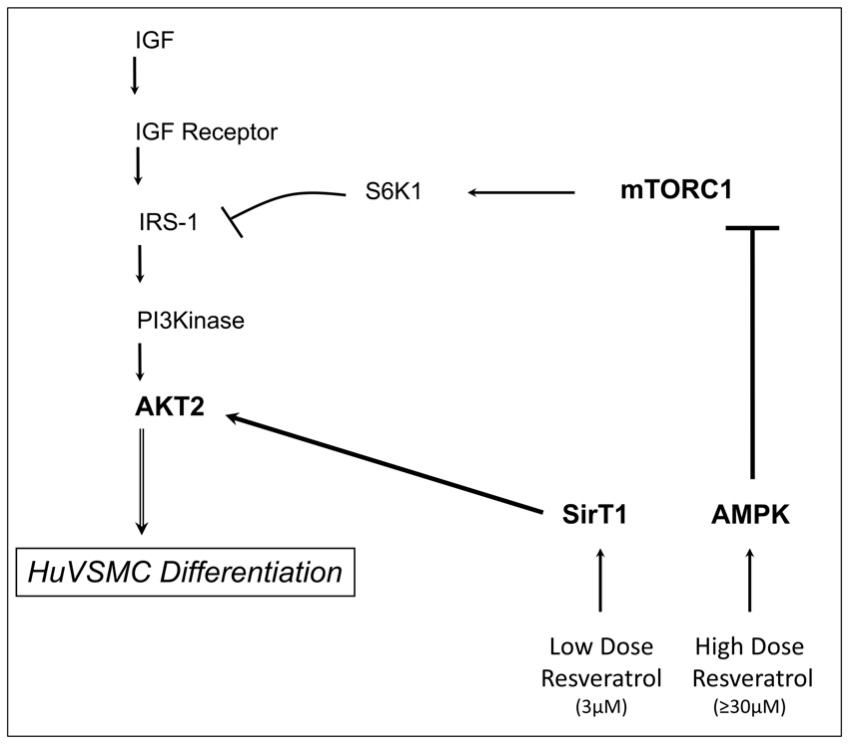
Model of Resveratrol Signaling in HuVSMC Differentiation. Resveratrol stimulates multiple second messengers that lead to AKT activation and the induction of differentiation. Low dose resveratrol stimulates SirT1, which activates AKT to induce differentiation. High dose resveratrol stimulates AMPK, which inhibits the mTORC1 pathway relieving the S6K1 inhibition on IRS-1, allowing activation of AKT and the induction of differentiation. Ultimately, resveratrol induces a multi-signaling cascade that results in robust vascular smooth muscle differentiation.

SirT1 and AMPK have a long history of co-existence and inter-dependence for activation. AMPK has been proposed to indirectly regulate SirT1 activity by altering NAD^+^ availability [9], [17]. Conversely, SirT1 deacetylation of LKB1, which promotes LKB1 translocation to the cytoplasm and phosphorylation of AMPK, is thought to directly regulate AMPK activity [20]. Ultimately, considering the shared roles AMPK and SirT1 play in basic energy metabolism it is no surprise that these molecules are fundamental in regulating vasculature remodeling through phenotypic modulation. Unique to our data, we demonstrate that both SirT1 and AMPK regulate vascular smooth muscle cell differentiation. The differentiation-signaling pathway elicited by resveratrol treatment capitalizes on the fact that both low dose, through SirT1 and high dose, through AMPK, elicit robust differentiation (Figure 6). The final messenger in either the SirT1 pathway or the AMPK pathway to differentiation is AKT; we have previously demonstrated that AKT2 activation is necessary for contractile protein expression [3]. SirT1 is known to promote membrane localization and activation of AKT via SirT1-dependent deacetylation of two lysine residues in the PH (pleckstrin homology) domain of AKT [21]. This SirT1-dependent deacetylation is necessary for AKT to bind to PIP_3_ and is necessary for AKT phosphorylation and, therefore, activation to occur. We have demonstrated that low dose resveratrol treatment increases phosphorylation of AKT and that knockdown of SirT1 eliminates activation of AKT (Figures 3 and 6). These combined data demonstrate that following low dose resveratrol treatment differentiation is induced through SirT1 direct activation of AKT; an activity that is independent of AMPK activation.

Furthermore, Ghosh et al. have demonstrated that SirT1 negatively regulates mTORC1 in mouse embryonic fibroblasts [22]: SirT1 associates with TSC2, and the interaction of SirT1 with TSC2 was demonstrated to be necessary for mTORC1 inhibition. Additionally, consistent with our present experiments, Ghosh et al. found a dose dependent effect of resveratrol on mTORC1 inhibition; Ghosh found that resveratrol treatment up to 25 µM stimulated SirT1 and negatively regulated the mTORC1 pathway, but high dose resveratrol treatment (100 µM), independent of SirT1, maintained mTORC1 inhibition. Consistent with our findings in Figure 4, we suggest that the resveratrol-induced SirT1-independent inhibition of the mTORC1 pathway is mediated by stimulation of AMPK. High dose resveratrol treatment therefore, induces contractile protein expression through AMPK activation and inhibition of the mTORC1 pathway, leading to activation of AKT and the induction of differentiation.

In this study, we have demonstrated that the polyphenol, resveratrol, that is heralded to prevent a broad range of diseases [23], can amplify a web of second messengers that leads to robust induction of vascular smooth muscle cell differentiation. This broader-multi-pathway stimulation allows for a rapid and robust induction of differentiation.

### Conclusions

Taken together, our experiments have demonstrated that resveratrol may be a unique drug for eliciting vascular smooth muscle cell differentiation; treatment with resveratrol activates multiple second messengers leading to differentiation. Ultimately, resveratrol treatment induces both SirT1 and AMPK and these molecules translate the vasculature crosstalk into activation of AKT and phenotypic modulation of the smooth muscle cells. Together, SirT1 and AMPK function as key coordinators of signaling pathways that regulate long term vasculature plasticity.

## Supporting Information

Figure S1 **Resveratrol Stimulates a Contractile Morphology.** HuVSMC were treated with 3 µM resveratrol or control for 24 hours. Cells were stained for SM2-MHC or SM-Alpha Actin (green) and the nucleus (blue). Images were captured on confocal microscope.
(TIF)

Figure S2 **Resveratrol Stimulates HuVSMC to Contract.** A, Representative images of contracted collagen discs treated with 3 µM resveratrol or control at 27 hours. Cells were pre-treated for 24 hours prior to being placed in collagen matrix. N = 2 experiments, in triplicate. B, HuVSMC were treated with 100 µM carbachol- a positive control of VSMC contraction, 3 µM resveratrol or control for 24 hours prior to measurement of contracted collagen disc. Bar graphs represent mean area change plus standard error of mean. N = 2 experiments, in triplicate. Probability values are indicated above bars: * p<0.05, versus control. C, HuVSMC treated with 5 µM resveratrol or control for 2 or 3 hours prior to measurement of contracted collagen disc, demonstrating as early as two hours post treatment, resveratrol stimulates contraction. Bar graphs represent mean area change plus standard error of mean. N = 2 experiments, in triplicate. Probability values are indicated above bars: * p<0.05, versus control.
(TIF)

Figure S3 **Resveratrol Activates AKT2, but not AKT1 or 3.** A, HuVSMC treated with 3 µM resveratrol or control for 24 hours prior to collection for immunoprecipitation (IP) with AKT2. Lysate was blotted back (BB) for activated AKT with antibody against phosphorylated AKT. B, HuVSMC treated with 3 µM resveratrol or control for 24 hours prior to collection for immunoprecipitation (IP) with AKT1 or AKT3. Lysate was blotted back (BB) for activated AKT with antibody against phosphorylated AKT.
(TIF)

Figure S4 **AKT Activation is Necessary for Low or High Dose Resveratrol-Induced Differentiation.** HuVSMC were treated with 20 µM API-2 one-hour prior to resveratrol or control treatment. Twenty-four hours following resveratrol treatment, cells were collected and assessed by western blot with primary antibodies as indicated. N = 2 experiments.
(TIF)

Figure S5 **AMPK is Necessary for High Dose Resveratrol-Induced Differentiation.** HuVSMC were transfected with control siRNA or AMPK α1 siRNA for 24 hours, prior to treatment with resveratrol or control. Twenty-four hours after treatment, cells were collected and assessed by western blot with primary antibodies as indicated. N = 2 experiments.
(TIF)
