# Supplementary figures and images for: Resveratrol Induces Vascular Smooth Muscle Cell Differentiation through Stimulation of SirT1 and AMPK
Source: PLoS One. 2014 Jan 8;9(1):e85495. doi: 10.1371/journal.pone.0085495 (PMC3885718; doi:10.1371/journal.pone.0085495)

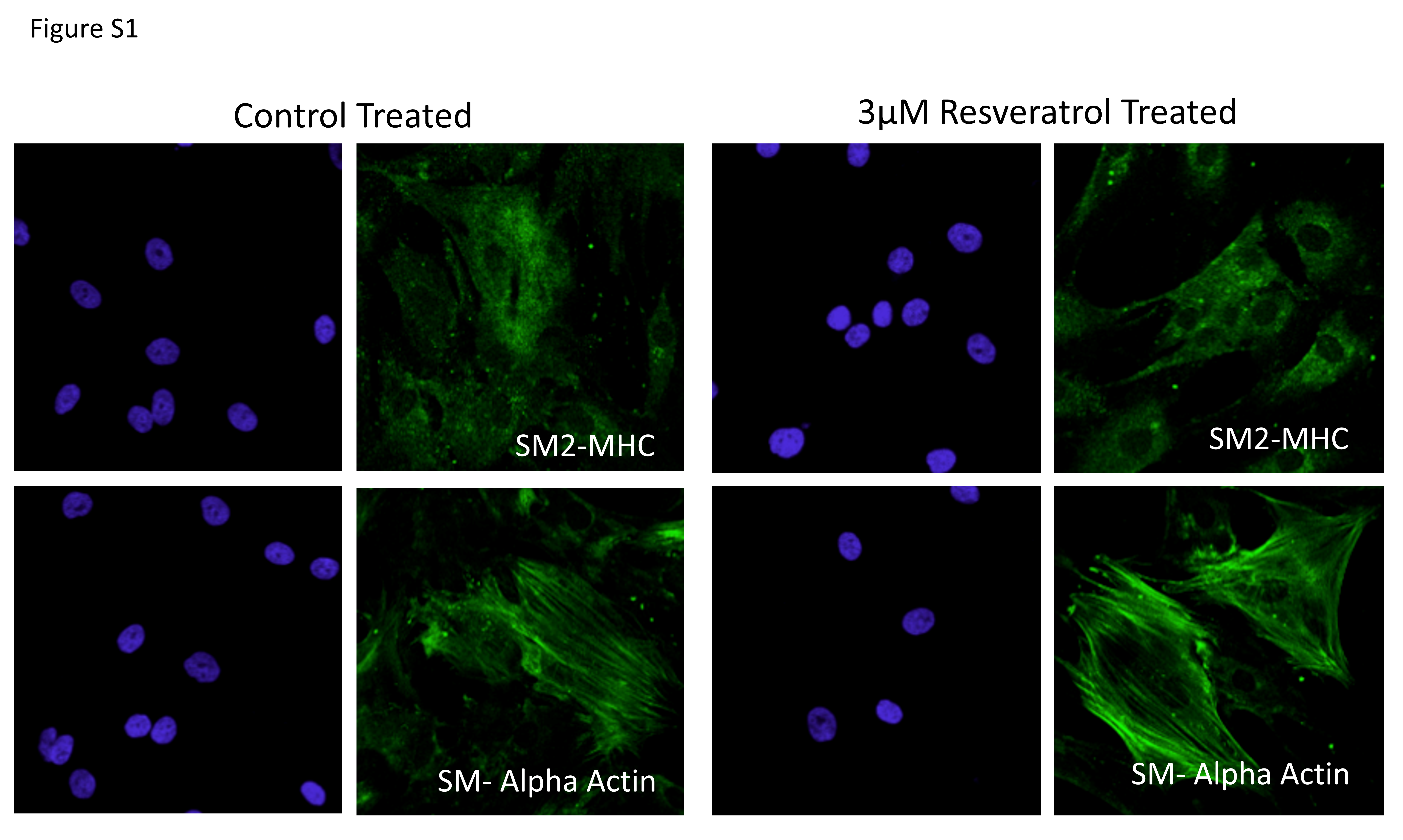

Supplement: Figure S1 — Resveratrol Stimulates a Contractile Morphology. HuVSMC were treated with 3 µM resveratrol or control for 24 hours. Cells were stained for SM2-MHC or SM-Alpha Actin (green) and the nucleus (blue). Images were captured on confocal microscope. (TIF) [file pone.0085495.s001.tif]

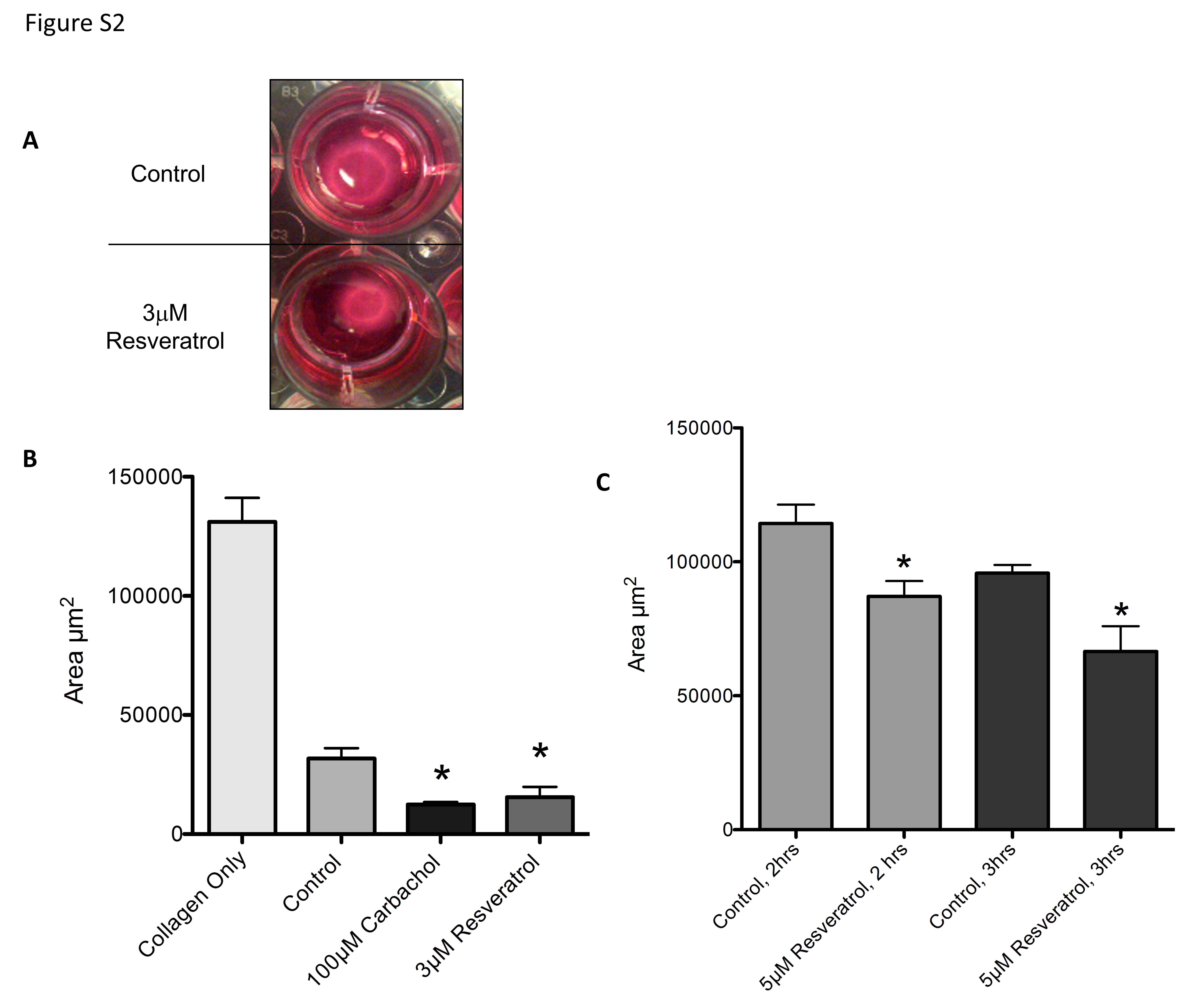

Supplement: Figure S2 — Resveratrol Stimulates HuVSMC to Contract. A, Representative images of contracted collagen discs treated with 3 µM resveratrol or control at 27 hours. Cells were pre-treated for 24 hours prior to being placed in collagen matrix. N = 2 experiments, in triplicate. B, HuVSMC were treated with 100 µM carbachol- a positive control of VSMC contraction, 3 µM resveratrol or control for 24 hours prior to measurement of contracted collagen disc. Bar graphs represent mean area change plus standard error of mean. N = 2 experiments, in triplicate. Probability values are indicated above bars: * p<0.05, versus control. C, HuVSMC treated with 5 µM resveratrol or control for 2 or 3 hours prior to measurement of contracted collagen disc, demonstrating as early as two hours post treatment, resveratrol stimulates contraction. Bar graphs represent mean area change plus standard error of mean. N = 2 experiments, in triplicate. Probability values are indicated above bars: * p<0.05, versus control. (TIF) [file pone.0085495.s002.tif]

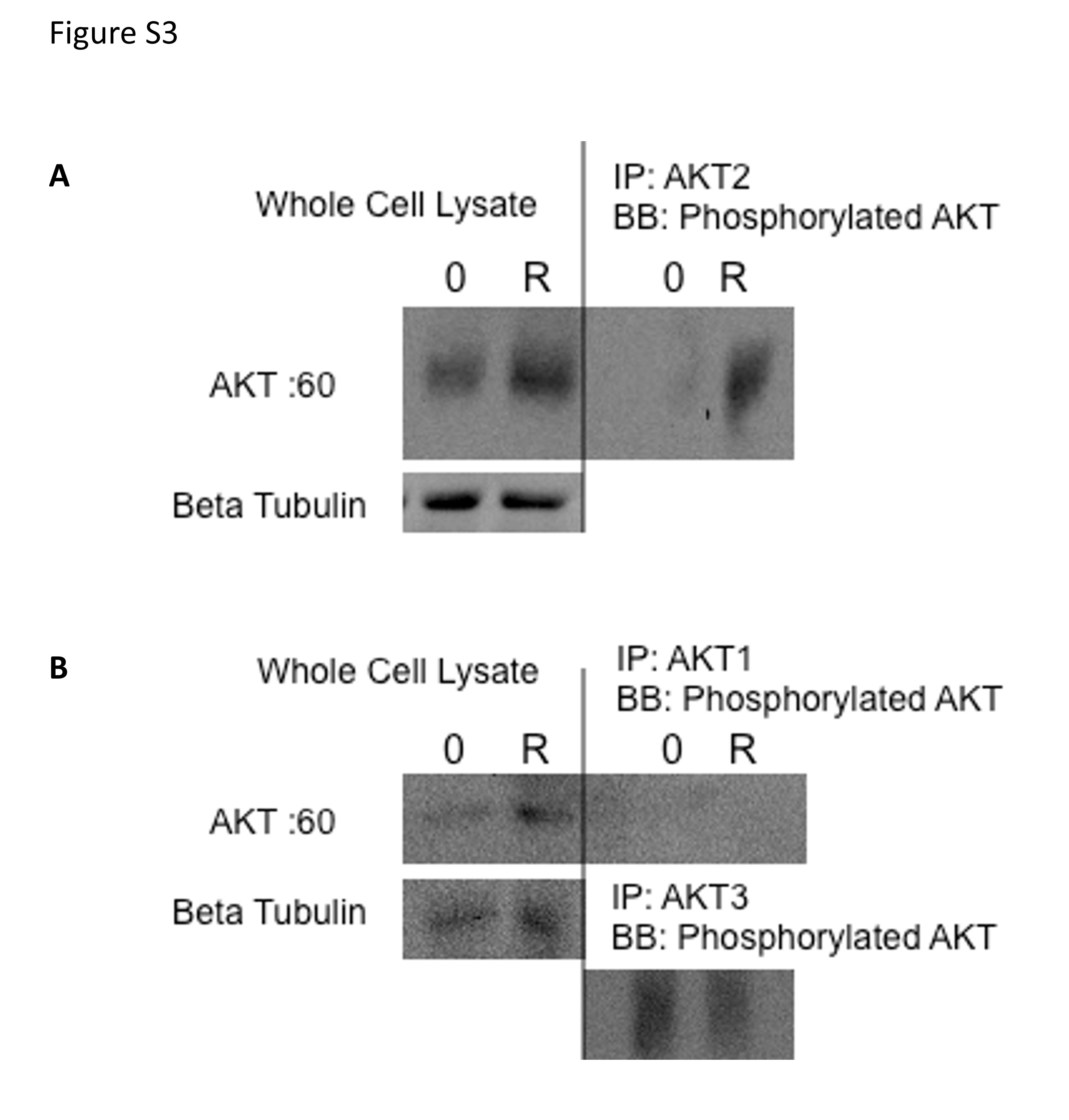

Supplement: Figure S3 — Resveratrol Activates AKT2, but not AKT1 or 3. A, HuVSMC treated with 3 µM resveratrol or control for 24 hours prior to collection for immunoprecipitation (IP) with AKT2. Lysate was blotted back (BB) for activated AKT with antibody against phosphorylated AKT. B, HuVSMC treated with 3 µM resveratrol or control for 24 hours prior to collection for immunoprecipitation (IP) with AKT1 or AKT3. Lysate was blotted back (BB) for activated AKT with antibody against phosphorylated AKT. (TIF) [file pone.0085495.s003.tif]

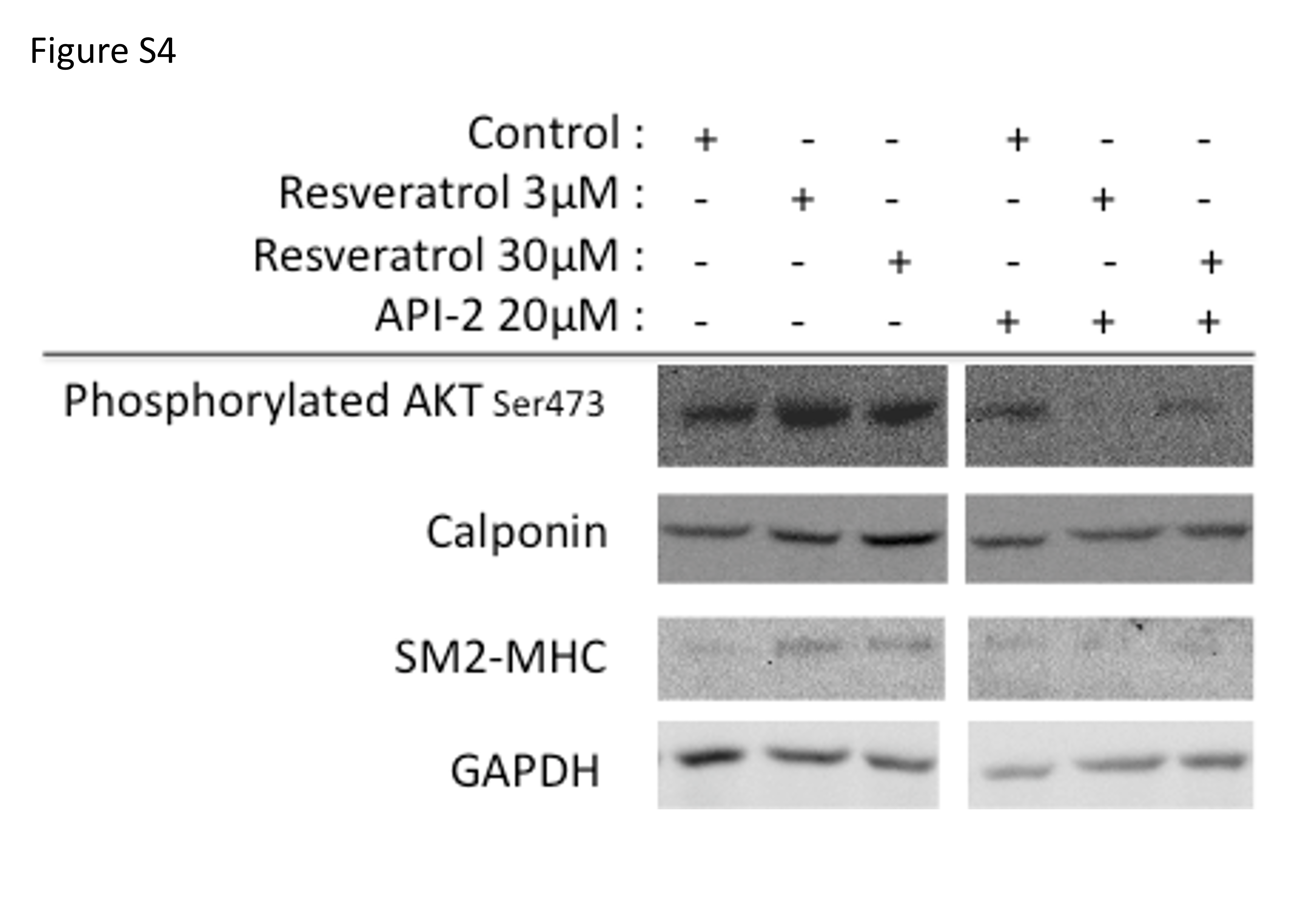

Supplement: Figure S4 — AKT Activation is Necessary for Low or High Dose Resveratrol-Induced Differentiation. HuVSMC were treated with 20 µM API-2 one-hour prior to resveratrol or control treatment. Twenty-four hours following resveratrol treatment, cells were collected and assessed by western blot with primary antibodies as indicated. N = 2 experiments. (TIF) [file pone.0085495.s004.tif]

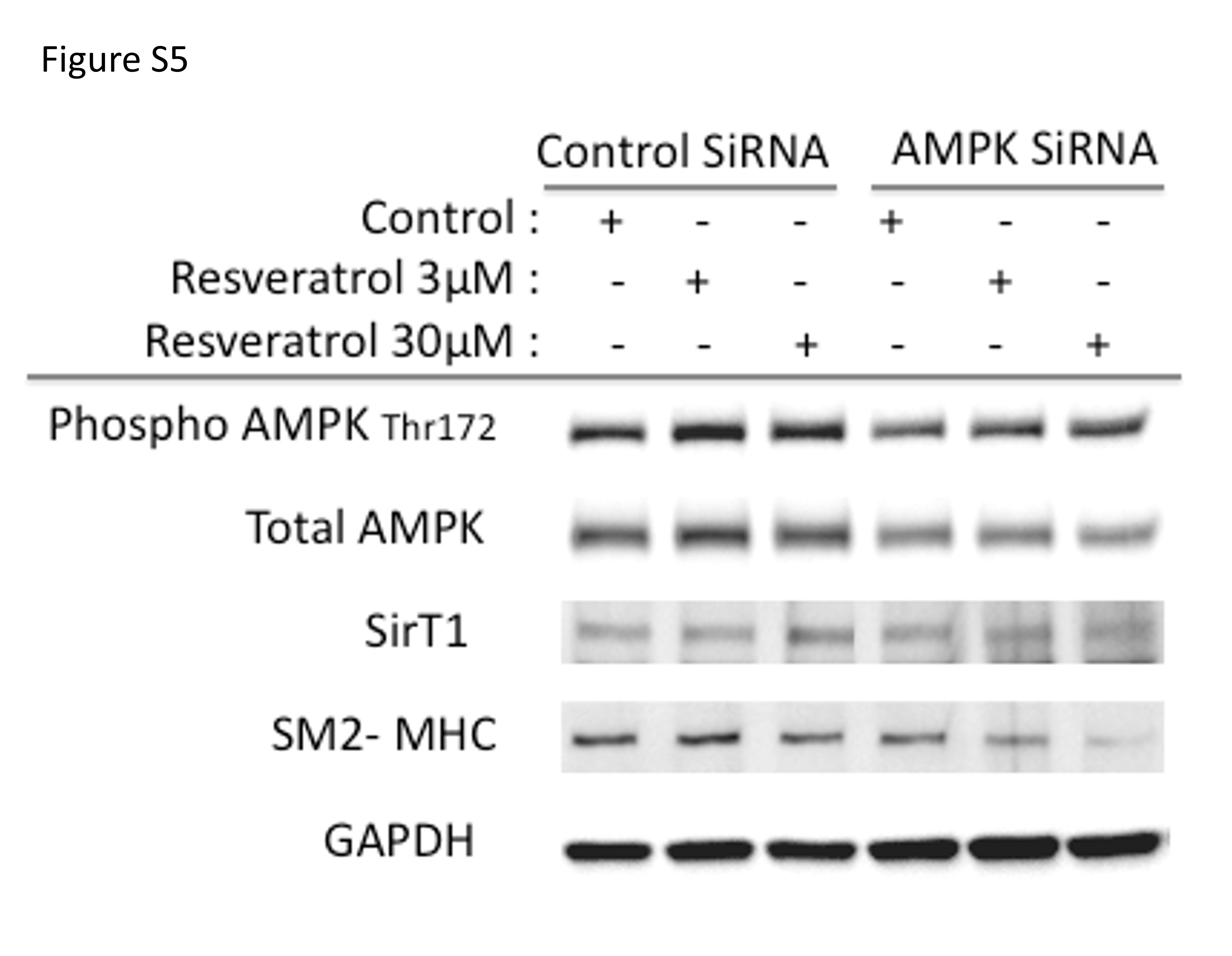

Supplement: Figure S5 — AMPK is Necessary for High Dose Resveratrol-Induced Differentiation. HuVSMC were transfected with control siRNA or AMPK α1 siRNA for 24 hours, prior to treatment with resveratrol or control. Twenty-four hours after treatment, cells were collected and assessed by western blot with primary antibodies as indicated. N = 2 experiments. (TIF) [file pone.0085495.s005.tif]
